# Supplementary material for: Protective Effect of Resveratrol against Ischemia-Reperfusion Injury via Enhanced High Energy Compounds and eNOS-SIRT1 Expression in Type 2 Diabetic Female Rat Heart
Source: Nutrients. 2019 Jan 6;11(1):105. doi: 10.3390/nu11010105 (PMC6356423; doi:10.3390/nu11010105)
Supplement: Supplementary file 1 [file nutrients-11-00105-s001.pdf]

Protective Effect of Resveratrol against Ischemia-Reperfusion Injury via Enhanced High Energy Compounds and eNOS-SIRT1 Expression in Type 2 Diabetic Female Rat Heart

Natacha Fourny, Carole Lan, Eric Sérée, Monique Bernard and Martine Desrois.

A

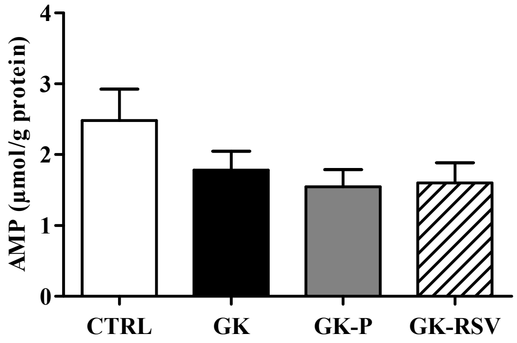

B

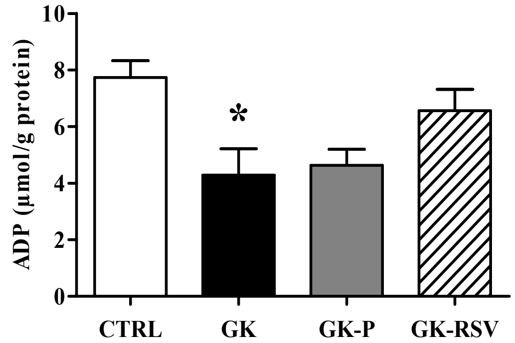

C

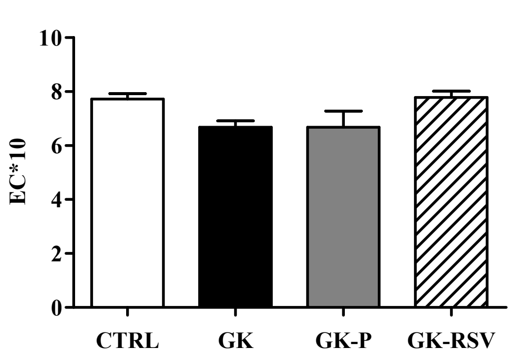

Figure S1. AMP (**A**), ADP (**B**) and EC*10 (**C**) in rat hearts. Data are expressed as means ± SEM and one-way ANOVA was used to compare the groups. No statistical difference was found between groups for AMP content. ADP content was significantly decreased only in GK versus CTRL (*$p<0.05$).

RSV increased ADP content but it did not reach statistical difference. No difference was found between groups for energy charge.

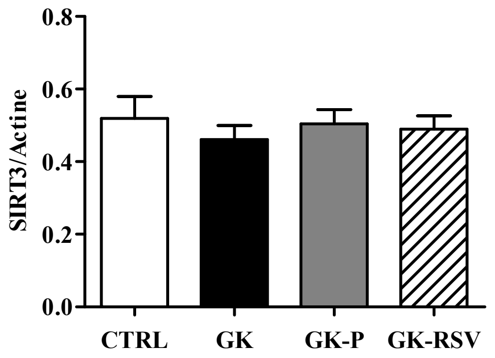

Figure S2. Protein expression of SIRT3 in rat hearts. Data are expressed as means ± SEM and one-way ANOVA was used to compare the groups. No statistical difference was found between groups.

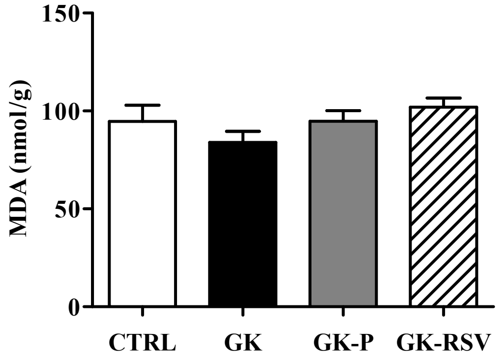

Figure S3. MDA heart content in rat hearts. Data are expressed as means ± SEM and one-way ANOVA was used to compare the groups. No statistical difference was found between groups.
